# *NtGCN2* mediates tobacco cold tolerance through chlorophyll retention, proline accumulation, antioxidant defense, and ABA regulation

**DOI:** 10.3389/fpls.2026.1833586

**Published:** 2026-05-08

**Authors:** Hang Li, Hao Wang, Dongling Wu, Jiao Du, Manman Zhang, Zhengyu Su, Yongxia Yang, Hongfang Jia, Litao Hu, Ning Li, Songtao Zhang

**Affiliations:** 1 National Tobacco Cultivation & Physiology & Biochemistry Research Centre, College of Tobacco Science, Henan Agricultural University, Zhengzhou, China; 2 Luoyang Branch of Henan Provincial Tobacco Company, Luoyang, China; 3 Fengdu Branch of Chongqing Tobacco Company, Chongqing, China; 4 College of Life Sciences, Henan Agricultural University, Zhengzhou, China

**Keywords:** cold stress, GCN2, molecular mechanisms, *Nicotiana tabacum*, RNA-seq

## Abstract

**Introduction:**

Cold conditions severely limit plant productivity. Accordingly, elucidating the physiological and biochemical changes and molecular mechanisms in plants under low-temperature stress is necessary. The conserved kinase general control non-derepressible 2 (GCN2) regulates stress responses; however, its role in cold adaptation remains elusive.

**Methods:**

Considering that tobacco (*Nicotiana tabacum* L.) is an ideal model plant for understanding the mechanisms through which economic crops respond to abiotic and biotic stress, this study aimed to elucidate the role and potential mechanisms of *NtGCN2* in the cold-stress response of tobacco plants. *NtGCN2*-overexpressing and wild-type K326 tobacco plants were exposed to low-temperature treatment at 4°C for 12 h. Thereafter, antioxidant activity was evaluated, abscisic acid (ABA) and chlorophyll content were measured, and transcriptomic profiling was conducted.

**Results:**

*NtGCN2*-overexpressing lines and K326 were subjected to low-temperature treatment. Integrated analyses revealed that *NtGCN2* coordinates multiple adaptive responses under cold stress. Specifically, *NtGCN2* preserved photosynthetic capacity by maintaining chlorophyll levels through upregulation of biosynthesis genes (e.g., *HEMA1* and *POR1*) and chloroplast-associated regulators. It promoted osmotic adjustment by enhancing proline accumulation via upregulation of biosynthesis and transport genes (e.g., *PAP1* and *ProT*) and nitrogen signaling components (*AMTs* and *NRTs*). Furthermore, *NtGCN2* alleviated oxidative damage by increasing superoxide dismutase and catalase activities and inducing peroxidase-related genes. In parallel, *NtGCN2* elevated ABA accumulation through upregulation of biosynthetic genes (e.g., *NCED1*) while attenuating ABA signaling components such as *COP1*. Collectively, these changes contributed to reduced oxidative damage (lower MDA levels) and improved stress resilience.

**Discussion:**

These findings suggest that *NtGCN2* functions as a central regulator of cold adaptation by integrating multiple physiological and molecular pathways, including photosynthetic maintenance, osmotic regulation, antioxidant defense, and ABA-mediated signaling. By modulating key downstream targets, *NtGCN2* enhances plant tolerance to low-temperature stress. This study expands the functional understanding of the conserved GCN2 signaling network in plants and highlights its potential as a genetic target for improving crop resilience to cold environments.

## 1 Introduction

Temperature is a key environmental factor in plant growth and development that enables plants to grow normally only under suitable temperatures. Among the various temperature stressors, low-temperature stress is one of the most common and detrimental, causing substantial annual yield losses in global crop production (Soualiou et al., 2022; Zhang et al., 2022). Therefore, elucidating the physiological and biochemical changes in plants under low-temperature stress and uncovering the molecular mechanisms underlying plant responses to cold stress are crucial for improving plant cold tolerance.

Low-temperature stress disrupts multiple physiological processes in plants. It impairs membrane fluidity and stability, leading to increased electrolyte leakage and cellular damage (Milovskaya et al., 2026). Photosynthesis is particularly sensitive to cold, with reduced chlorophyll content, inhibited enzyme activities, and impaired chloroplast structure resulting in decreased photosynthetic efficiency (Sati et al., 2025; Sardoei and Fazeli-Nasab, 2025). In addition, cold stress induces the overproduction of reactive oxygen species (ROS), causing oxidative damage to lipids, proteins, and nucleic acids (Rao et al., 2025). To cope with these challenges, plants often accumulate osmoprotectants such as proline and soluble sugars to maintain cellular osmotic balance and protect macromolecular structures.

Over the course of evolution, plants have developed sophisticated systems to perceive and transduce low-temperature signals, thus enabling them to sense environmental temperature fluctuations rapidly and actively mitigate cold-induced damage (Zhang et al., 2022; Jahed et al., 2023).

Plants perceive low-temperature stress through changes in cellular membranes and photoreceptors. The fluidity of the plasma membrane is closely linked to the ratio of unsaturated fatty acids and plays a pivotal role in cold perception (Kargiotidou et al., 2008). Fatty acid desaturase 2 (*FAD2*) encodes an oleate desaturase that affects membrane fluidity. Moreover, *fad2* mutants exhibit altered sensitivity to cold stress. Therefore, membrane fluidity is a key component of cold sensing in plants (Chen and Thelen, 2013). Under low-temperature stress, malondialdehyde (MDA) content is negatively correlated with cold tolerance and serves as an indicator of reactive oxygen species (ROS)-induced membrane damage within plant tissues (Sato et al., 2011). The photoreceptor state facilitates cold-signal transmission. Upon exposure to cold, photoreceptors, such as phytochrome A (phyA) and phytochrome B, shift from their active Pfr form to the inactive Pr form. This consequently affects photomorphogenesis and photosynthesis (Wang et al., 2015; Paik and Huq, 2019). Moreover, exposure to low-temperature stress induces a substantial increase in endogenous abscisic acid (ABA) levels, especially in the root and leaf tissues, where rapid accumulation is often observed (Guo et al., 2018). Once cold stress is perceived, ABA acts as a key signaling molecule to activate a cascade of downstream adaptive responses at the molecular and physiological levels (Sato et al., 2011; Zhang et al., 2022).

Upon perception of cold signals, plants transduce these signals via secondary messengers, such as calcium ions (Ca²^+^) and ROS. Ca²^+^ is a key signaling molecule in plant responses to environmental stimuli, and cold-induced Ca²^+^ influx is an early response to low-temperature stress (Zhang et al., 2022). Furthermore, the expression of specific cold-responsive (*COR*) genes is Ca²^+^-dependent (Iqbal et al., 2022). For instance, cyclic nucleotide-gated channels (*CNGC*) in *Arabidopsis* and moss and the chilling tolerance divergence 1 (COLD1)-regulator of G-protein signaling 1 (RGA1) complex in rice, which comprises the plasma membrane and endoplasmic reticulum-localized cold sensor COLD1 and RGA1, are all involved in regulating Ca²^+^ signaling under cold stress. This ultimately activates the expression of downstream COR genes (Shi and Yang, 2015; Iqbal et al., 2022). In addition to Ca²^+^, ROS function as signaling molecules during cold-stress responses (Mittler et al., 2022). However, the role of ROS is dual-faceted. Specifically, although moderate ROS levels activate gene expression and protein synthesis to protect cells, excessive ROS accumulation leads to oxidative stress (Xia et al., 2015).

Following signal perception and transduction, plants activate downstream transcriptional regulators and induce expression of *COR* genes. One of the best-characterized pathways involves C-repeat-binding factors (CBF)/dehydration-responsive element-binding (DREB) factors, which are rapidly induced by cold and bind to the promoters of *COR* genes to activate their transcription (Liu et al., 2018). Cold signaling pathways are typically categorized into *CBF/DREB*-dependent and *CBF/DREB*-independent pathways. In the *CBF/DREB*-dependent pathway, signal transduction proceeds primarily via the *ICE1–CBF–COR* module. Although the *CBF–COR* pathway is considered a central component of the plant cold response, many COR genes are not regulated by *CBFs* (Shi et al., 2018). Furthermore, transcription factors, such as *HSFC1, ZAT10/12, RAV1, CZF1*, and *HY5*, modulate plant cold tolerance independent of *CBFs* (Vogel et al., 2005; Catalá et al., 2011; Jia et al., 2016; Zhao et al., 2016; Fan et al., 2021).

In plants, general control non-derepressible 2 (GCN2) is the only known eukaryotic initiation factor 2α (eIF2α) kinase encoded by a single gene (Zhang et al., 2003, 2008). During protein synthesis, GCN2 phosphorylates the α-subunit of eIF2, thereby facilitating its binding to eIF2B (Zhang et al., 2008). This interaction prevents the conversion of eIF2γ-guanosine diphosphate to eIF2γ-guanosine triphosphate due to the lack of eIF2B. This disrupts the translation initiation cycle and regulates protein synthesis (Li et al., 2018). In addition, GCN2 is a protein kinase that responds to various stresses, including cold, drought, hormone treatments (SA and MeJA), herbicides (chlorsulfuron), heavy metals (cadmium), wounding, and ultraviolet radiation (Lageix et al., 2008; Li et al., 2018; Zhao et al., 2018; Liu et al., 2019; Llabata et al., 2019; Lokdarshi et al., 2020; Cho et al., 2022). Moreover, GCN2 upregulates the phosphorylation of its substrate NteIF2α and improves plant tolerance to drought and cadmium stress (Wang et al., 2023; Shi et al., 2024).

In *Arabidopsis thaliana*, AtGCN2 interacts with AtGCN1 to activate the phosphorylation of eIF2α, thereby enhancing *Arabidopsis* tolerance to cold stress (Wang et al., 2017). Moreover, AtGCN2 is essential for eIF2α phosphorylation and improving cold tolerance in *Arabidopsis*. As a homolog of AtGCN2, NtGCN2 may play an important role in the response of tobacco plants to cold stress. Tobacco is an ideal model plant that has long served as a valuable reference for understanding the mechanisms through which economic crops respond to abiotic and biotic stress. Therefore, to investigate the function of *NtGCN2* under cold stress, this study subjected *NtGCN2*-overexpressed(*NtGCN2*-OE) and K326 tobacco plants (*Nicotiana tabacum* L.) to low-temperature treatment.

Interactions between ROS and Ca²^+^ have been reported; however, the precise mechanisms of their crosstalk in cold signal transduction remain unclear. Furthermore, although the *CBF/DREB-*dependent signaling pathway has been extensively studied and is relatively well understood, the mechanisms and gene regulatory networks involved in the *CBF/DREB*-independent pathway remain largely unclear and warrant further investigation.

Therefore, this study aimed to elucidate the role and potential mechanisms of *NtGCN2* in the cold-stress response of tobacco plants by integrating physiological, biochemical, and RNA sequencing (RNA-seq) data. This study provides a theoretical basis for the identification of gene resources related to cold stress and contributes to understanding the signaling networks involved in cold-stress responses.

## 2 Materials and methods

### 2.1 Plant materials, growth conditions and stress treatments

The experimental materials, *Nicotiana tabacum* L. cv. K326 and three distinct *NtGCN2-*overexpressed tobacco lines (OE-1, OE-2, and OE-3), derived from the K326 transformation background, were developed and maintained in our laboratory (Li et al., 2018). In this study, K326 is widely adopted due to its stable genetic background and high transformation efficiency, which facilitate transgenic analysis. Moreover, its moderate sensitivity to abiotic stresses, including cold stress, makes it a suitable model system for assessing gene function and dissecting molecular mechanisms underlying stress tolerance (Din et al., 2025).

The seeds were disinfected using a 10% sodium hypochlorite solution, and then, with the aid of a toothpick, the disinfected seeds were sown on MS solid medium in a sterile laminar flow hood After 10 days of continuous cultivation, the tobacco seedlings were transferred to black square pots (10 × 10 cm) containing a mixture of sterile soil and nutrient soil (Floragard, Germany) in a 1:1 ratio. They were grown under normal watering conditions for 3–4 days to allow acclimatization, and then uniformly growing plants were selected for stress treatments. The seeds and transplanted tobacco plants were placed in a growth chamber under light conditions of 28°C with a 16 h/8 h light/dark cycle and a relative humidity of (75 ± 5)%.

For cold stress, tobacco seedlings were subjected to 4°C for 0, 3, and 6 hours (under continuous light conditions). At each time point, the third leaf from the top was collected, with three independent biological replicates at each time point. The samples were flash-frozen in liquid nitrogen and stored at -80°C for subsequent quantitative analysis and RNA-seq. Samples for physiological and biochemical analysis were collected at 0, 3, 6, and 12 hours of cold stress treatment. For germination rate experiments, seeds were subjected to 15°C low-temperature stress to avoid the inhibitory effect of 4°C on seed germination.

### 2.2 Measurement of chilling injury index

Chilling injury (CI) was assessed visually based on the proportion of leaf surface area exhibiting pitting and dark, water-soaked lesions. The severity of CI was rated on a 5-point scale as follows: 0 = no visible injury; 1 = injury affecting < 25% of the surface area; 2 = 25–50%; 3 = 51–75%; and 4 = > 75%. The CI index was calculated using the following formula: CI index (between 0 and 4) = [∑ (CI scale × number of leaf at each CI level)/total number of leaf in the treatment] (Huqing Yang, 2011).

### 2.3 Germination rate and phenotypic characterization

Low-temperature stress (≤ 15°C) inhibits the germination of tobacco seeds, and as the temperature decreases further, the germination time is progressively prolonged, with seeds potentially failing to germinate altogether. Therefore, a temperature of 15°C was selected for the tobacco seed germination experiment (Gechev et al., 2003; Wang, 2006). A layer of sterile absorbent cotton was placed at the bottom of a circular glass Petri dish, followed by a sheet of filter paper. The sterile absorbent cotton and filter paper were thoroughly wetted with water. Disinfected seeds were then placed on the filter paper at a 10 × 10 cm ratio using a toothpick. The Petri dishes were subsequently placed in a light-controlled growth chamber set to 15°C and 25°C for seed germination experiments, with three biological replicates for each genotype.

Phenotypic images of tobacco seedlings subjected to cold stress were taken and analyzed. Different cold stress symptoms, such as leaf color, morphology, wilting, and dehydration, were classified and rated according to severity (The cold damage index was calculated independently for each plant.).

### 2.4 Histochemical staining

The superoxide anion (O_2_^-^) was detected in tobacco seedlings by conducting histochemical staining with nitroblue tetrazolium (NBT) (Kong et al., 2011). Tobacco seedlings or punched leaf discs were immersed in NBT staining solution for 1 hour, followed by substitution with 95% ethanol. After decolorization in the dark at 37°C, images were taken.

### 2.5 Determination of antioxidant enzyme activity

Superoxide Dismutase (SOD) and Catalase (CAT) were measured using an enzyme activity assay kit (Keming Biotechnology Co., Ltd.).

### 2.6 Determination of malondialdehyde, proline and ABA content

MDA and free proline were determined using MDA and proline assay kits (Nanjing Zhongding Biotechnology Co., Ltd., China).

ABA content was determined using an enzyme-linked immunosorbent assay (ELISA; China Agricultural University, Beijing, China).

### 2.7 Determination of chlorophyll content

As described by previous studies, a certain mass of tobacco leaves was immersed in 95% ethanol for 24 hours. The absorbance of the supernatant was measured using a UV spectrophotometer, and chlorophyll content was calculated based on the absorbance values (Wang, 2006; Wang et al., 2023).

### 2.8 RNA-seq analysis

Simultaneously subject 36 tobacco seedlings to 4°C cold stress, the third leaf (counted from the top) of tobacco seedlings was collected at 0 h, 3 h, and 6 h. The samples were immediately placed into cryogenic tubes, rapidly frozen in liquid nitrogen, and subsequently shipped on dry ice to OE Biotech Co., Ltd. (Shanghai, China) for further analysis. Each sample included three biological replicates, resulting in a total of 36 samples. The reference genome of *Nicotiana tabacum* cultivar K326, version Nitab4.5, was obtained from the Sol Genomics Network database (SGN; https://solgenomics.net/) (Edwards et al., 2017). Total RNA was extracted using TRIzol and quality-checked with NanoDrop 2000 and Agilent 2100. Libraries were constructed using the VAHTS Universal V5 kit and sequenced by OE Biotech (Shanghai, China). Clean reads (filtered by fastp) were aligned to the *Nicotiana tabacum* genome using HISAT2. Gene expression was quantified with HTSeq-count and normalized as FPKM. Differentially expressed genes (DEGs) were identified using DESeq2 (fold change > 2, q-value < 0.05), followed by GO and KEGG enrichment analyses (Wang et al., 2023).

### 2.9 Real-time quantitative reverse transcription PCR

Total RNA was extracted, and first-strand cDNA was synthesized as previously described. qRT-PCR was conducted using the same methodology (Li et al., 2018). The *NtL25* (L18908) gene was used as reference gene. The samples used for qRT-PCR validation of RNA-seq results were derived from another batch of independent replicate experiments. Primer design for all genes was carried out using Primer 5.0 and TBtools, and the corresponding sequences are provided in **Supplementary Table S1**.

## 3 Result

### 3.1 *NtGCN2* positively regulates cold tolerance in tobacco

**Figure 1:**
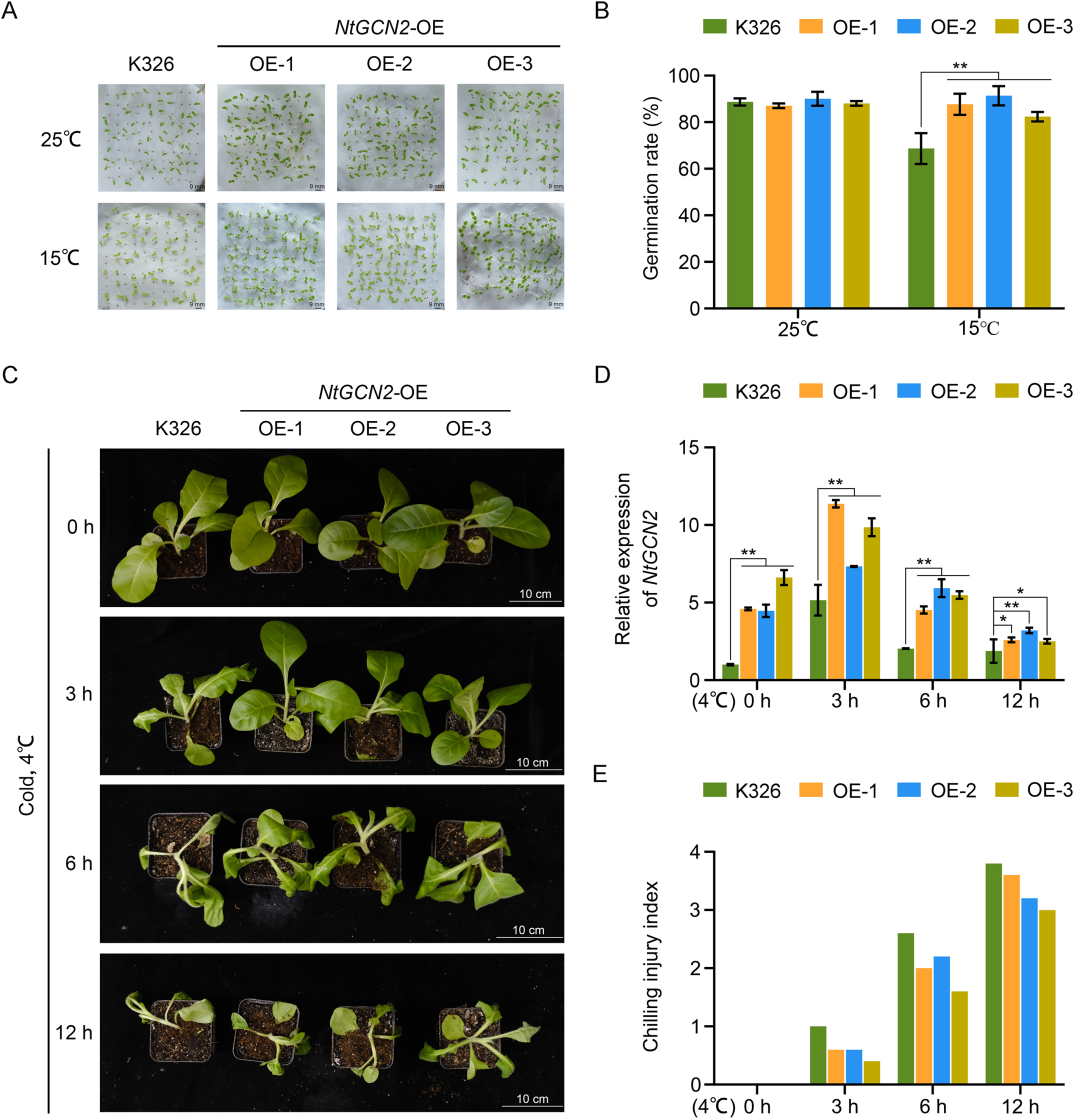
Cold-tolerance analysis of *NtGCN2*. **(A)** Photographs showing the maximum germination rate of transgenic and K326 tobacco seeds under low-temperature stress (15°C); **(B)** Quantification of germination rates at 15°C; **(C)** Phenotypes of transgenic and WT tobacco plants under low-temperature stress (4°C) for different durations; **(D)** Quantitative reverse transcription PCR (qRT-PCR) analysis of *NtGCN2* expression in transgenic and K326 plants under cold treatment; **(E)** Statistical analysis of damage severity in transgenic and WT plants under cold stress. For panel **(A)**, 100 seeds were used per plate in each experiment. Error bars represent the mean ± standard deviation (SD). Asterisks indicate statistically significant differences compared with those of the WT (*P* < 0.05 or *P* < 0.01), as determined by Duncan’s multiple range test.

Under control conditions (25°C), the germination rates of *NtGCN2*-overexpressed (OE) lines and K326 were comparable. However, under low-temperature stress (15°C), *NtGCN2*-OE lines exhibited markedly enhanced seed germination rates compared with those of K326 (**Figures 1A, B**).

At the beginning of the cold stress of seedlings, the *NtGCN2*-OE lines and K326 plants displayed similar phenotypes. In contrast, after exposure to cold stress, OE lines showed markedly reduced wilting symptoms relative to those of K326, thus demonstrating enhanced tolerance to cold stress. This phenotypic advantage was particularly evident during the initial 3 or 6 h of cold exposure (**Figures 1C, E**).

Furthermore, qRT-PCR analysis confirmed that *NtGCN2* expression was rapidly induced by cold treatment (**Figure 1D**). Collectively, these results demonstrate that *NtGCN2* is involved in the cold stress response of tobacco and that its overexpression enhances cold stress tolerance.

### 3.2 *NtGCN2* enhances ROS scavenging via antioxidant enzymes under cold stress

**Figure 2:**
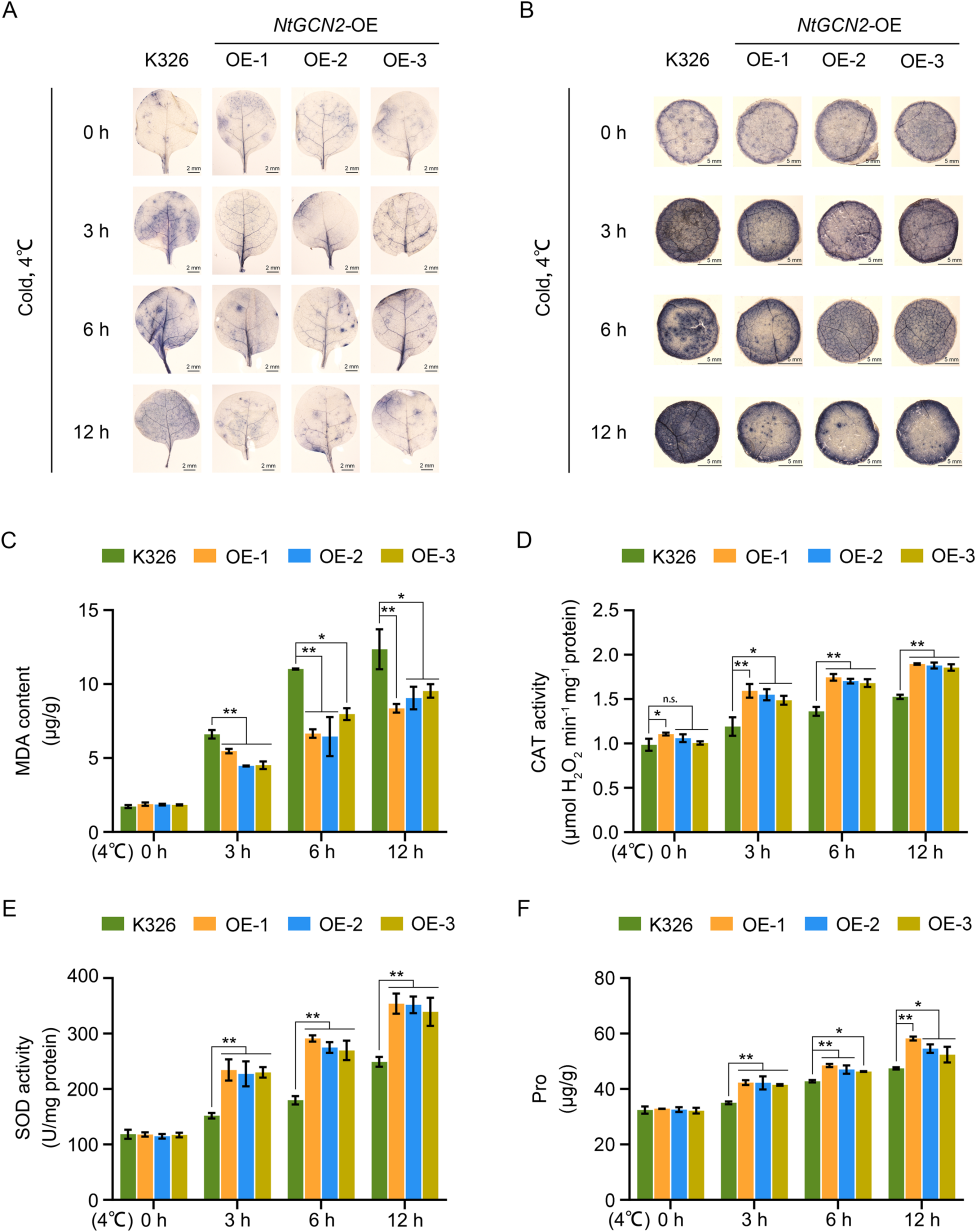
Antioxidant capacity of transgenic tobacco under cold stress. **(A)** Whole-leaf nitroblue tetrazolium (NBT) staining of two-leaf stage transgenic and K326 seedlings after cold treatment for different durations; **(B)** NBT staining of leaf discs (punched using a hole puncher) from 5–6 leaf stage seedlings after cold treatment; **(C–F)** Quantification of physiological and biochemical indices after cold treatment in transgenic and WT plants, including: **(C)** Malondialdehyde (MDA) content, **(D)** catalase (CAT) activity, **(E)** superoxide dismutase (SOD) activity, and **(F)** free-proline content. Error bars represent the mean ± standard deviation (SD) of three independent biological replicates (n = 3). Asterisks indicate statistically significant differences compared with those of the K326 (*P* < 0.05 or *P* < 0.01), as determined by Duncan’s multiple range test.

Low-temperature stress disrupts the equilibrium between ROS production and scavenging, leading to oxidative damage. To assess ROS accumulation, nitroblue tetrazolium (NBT) staining was performed to visualize superoxide anions (O_2_^-^) in the leaves of OE and K326 plants. Under control conditions, all lines exhibited comparable NBT staining intensity, thereby indicating similar basal O_2_^-^ levels. However, following cold stress, staining intensity increased in both genotypes, but this increase was substantially attenuated in OE lines compared with that of K326 plants, thereby demonstrating reduced O_2_^-^ accumulation (**Figures 2A, B**).

Excessive ROS accumulation induces lipid peroxidation, as quantified by MDA, a well-established biomarker of membrane damage. Consistent with the ROS data, the MDA content was similar across genotypes under control conditions. However, after cold treatment, the OE lines exhibited markedly lower MDA levels than those of K326, confirming reduced oxidative damage (**Figure 2C**).

To elucidate the mechanism underlying reduced ROS accumulation, the activities of the key antioxidant enzymes superoxide dismutase (SOD) and catalase (CAT) were measured. Under non-stress conditions, SOD and CAT activities were comparable between the OE lines and K326 plants. Strikingly, upon cold exposure, both SOD and CAT activities were considerably elevated in the OE lines relative to those in K326 (**Figures 2D, E**).

To evaluate osmotic adjustment, the leaf free-proline content was measured. Under cold stress, the OE lines accumulated markedly higher proline levels than those of K326, suggesting a potential role of *NtGCN2* in osmotic regulation under low-temperature conditions (**Figure 2F**).

Collectively, these results demonstrate that *NtGCN2* overexpression potentiates the antioxidant defense system under cold stress, thereby enhancing ROS detoxification through the upregulation of SOD and CAT activities, and ultimately mitigating oxidative damage.

### 3.3 *NtGCN2* modulates ABA accumulation and maintains chlorophyll content under cold stress

**Figure 3:**
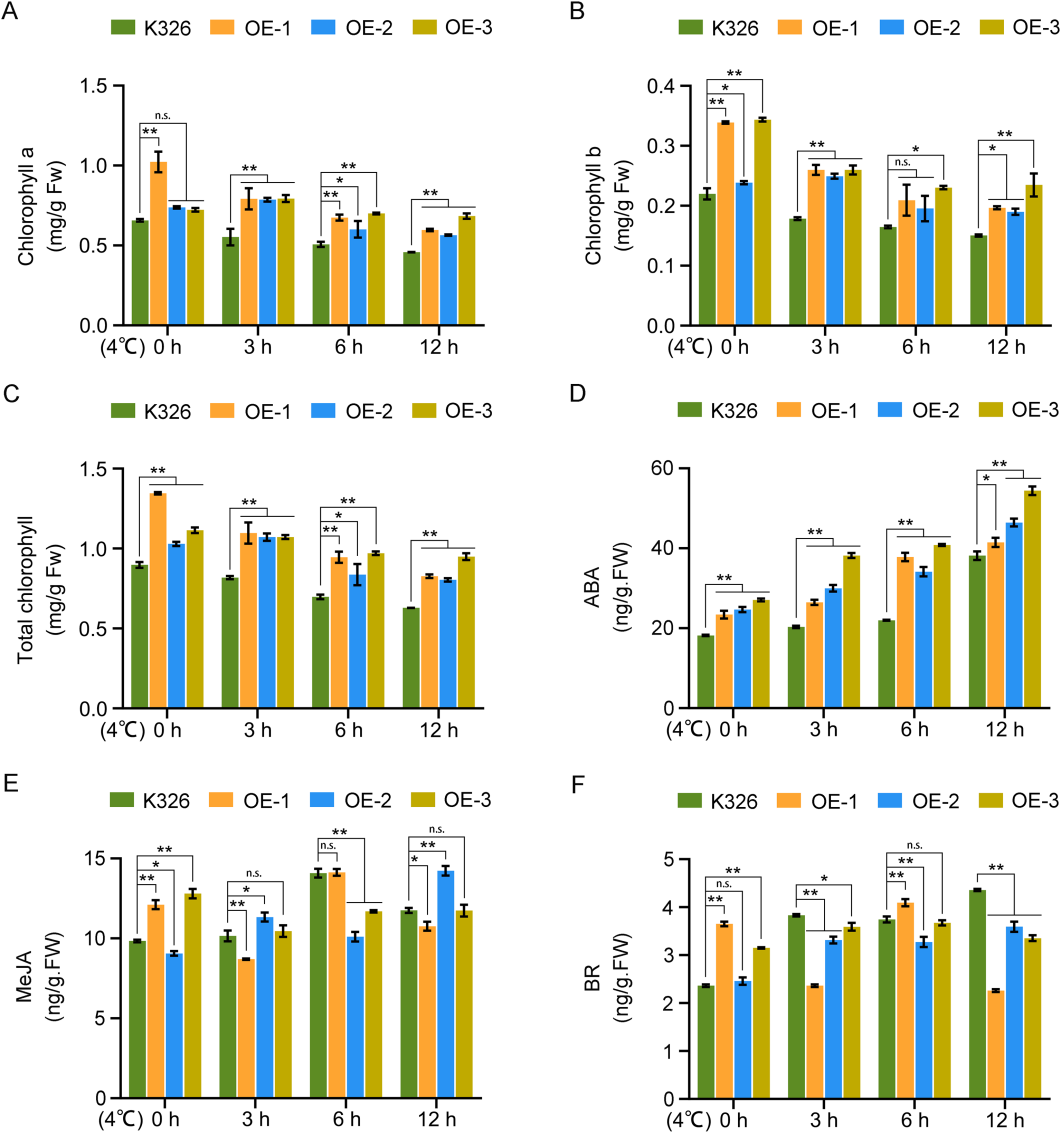
Chlorophyll and hormone contents in transgenic tobacco under cold conditions. **(A)** Chlorophyll a content; **(B)** Chlorophyll b content; **(C)** Total chlorophyll content; **(D)** Abscisic acid (ABA) content; **(E)** Methyl jasmonate (MeJA) content; **(F)** Brassinosteroid (BR) content. Error bars represent the mean ± standard deviation (SD) of three independent biological replicates (n = 3). Asterisks indicate statistically significant differences compared with those of K326 (*P* < 0.05 or *P* < 0.01), as determined by Duncan’s multiple range test.

The chlorophyll dynamics, which are critical indicators of photosynthetic competence, were monitored. Under control conditions, OE lines displayed elevated chlorophyll a/b, and total chlorophyll content relative to that in K326 plants. Following cold stress, all genotypes experienced chlorophyll degradation; however, the OE lines retained considerably higher chlorophyll levels than those in K326 plants (**Figures 3A–C**). This demonstrated that *NtGCN2* overexpression mitigated cold-induced chlorophyll loss, most likely by preserving photosynthetic capacity under stress conditions.

Given the established role of ABA in stress adaptation, ABA levels in OE and K326 plant were quantified during cold exposure. ABA content progressively increased with cold treatment duration in both genotypes. Notably, the OE lines exhibited markedly higher ABA accumulation than that in K326 plants at all time points (**Figure 3D**). However, MeJA and brassinosteroid levels were also measured, and no consistent patterns of change were observed (**Figures 3E, F**). These results indicate that *NtGCN2* positively regulates cold-induced ABA biosynthesis, potentially enhancing stress signaling.

### 3.4 Transcriptomic profiling reveals *NtGCN2*-mediated gene regulation under cold stress

Transcriptomic analysis of OE and K326 plants was conducted under cold stress. High-quality RNA sequencing of the 36 samples yielded an average of 48.91 million clean reads per sample (total = 1760.73 million reads; 253.58 Gb clean bases), with valid bases exceeding 94.15%, Q30 scores >93.93%, and GC content between 43.47–44.52% (**Supplementary Tables S2**-**S3**). Rigorous quality assessment, including principal component analysis and sample clustering, confirmed the high data reproducibility and reliability of the downstream analysis (**Supplementary Figure S1**).

**Figure 4:**
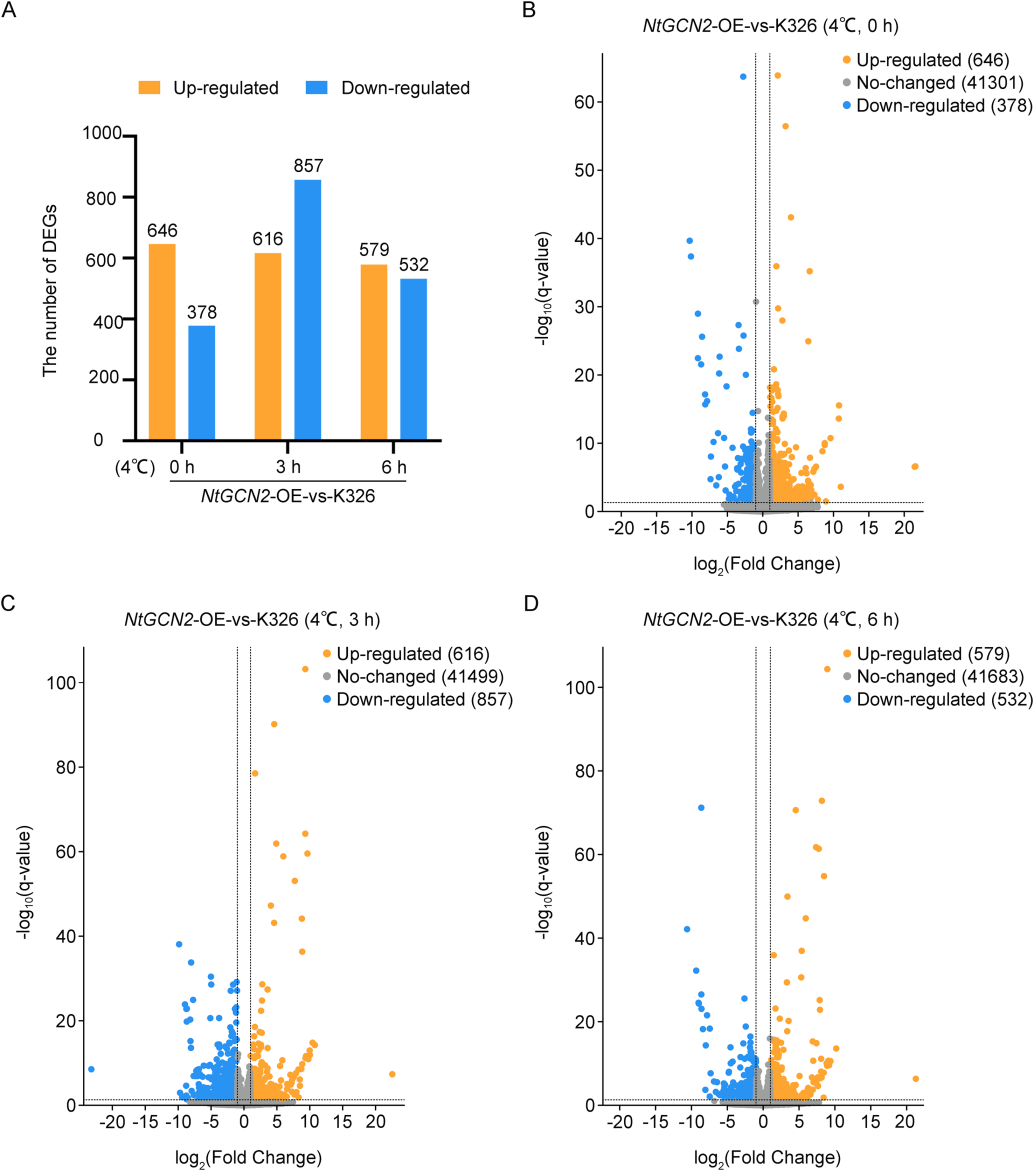
Transcriptomic analysis of *NtGCN2*-overexpressing (OE) plants compared to K326 under cold stress conditions. **(A)** Number of differentially expressed genes (DEGs) between *NtGCN2*-OE and K326 at 0 h, 3 h, and 6 h after cold treatment. DEGs were categorized into up-regulated and down-regulated genes. **(B–D)** Volcano plots showing DEGs at 0 h **(B)**, 3 h **(C)**, and 6 h **(D)**. Orange and blue dots represent significantly up- and down-regulated genes, respectively (|log_2_FC| > 1 and q-value < 0.05). Grey dots indicate genes with no significant change. Numbers of DEGs and non-DEGs are shown in the legend.

Differential gene expression revealed that under control conditions (25°C), 1024 differentially expressed genes (DEG) were identified between *NtGCN2*-overexpressing lines (OE) and K326 (**Figures 4A, B**). Following cold stress (4°C), DEG numbers increased to 1473 at 3 h and 1111 at 6 h (**Figures 4A, C, D**), thereby demonstrating that *NtGCN2* overexpression substantially reshapes the transcriptional response to cold.

**Figure 5:**
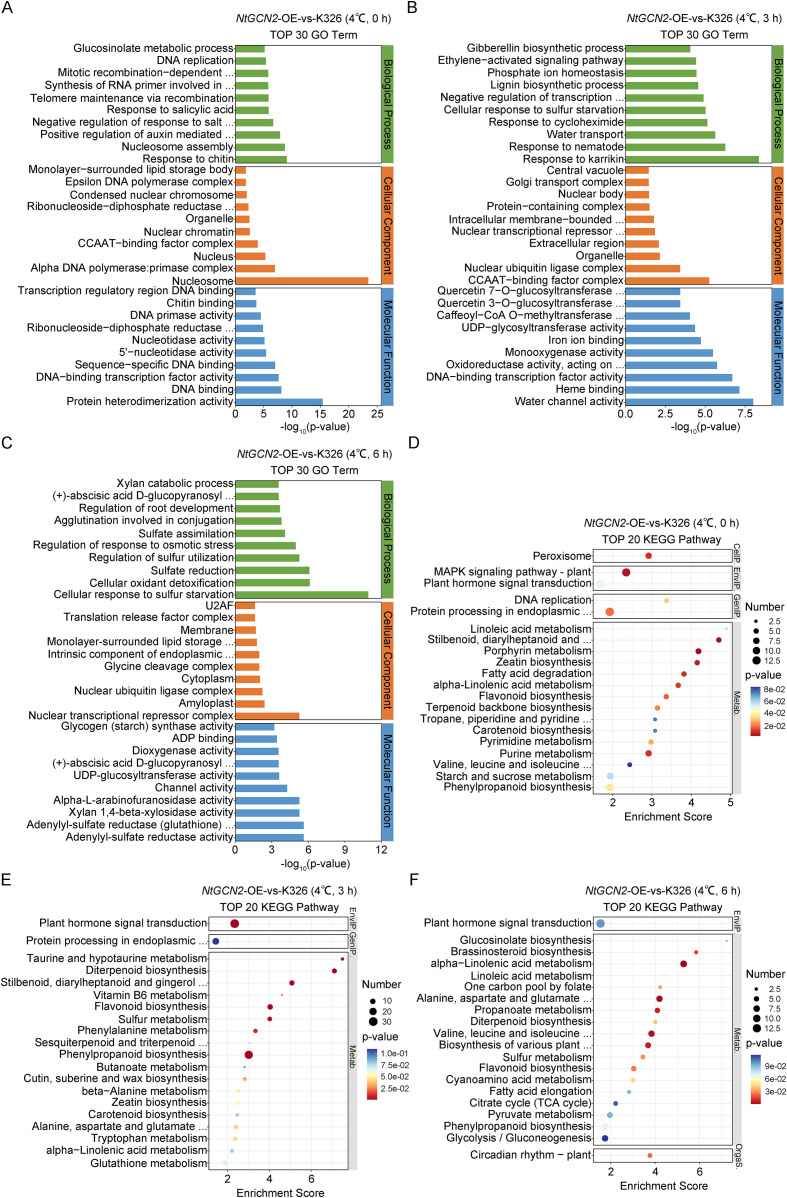
Gene ontology (GO) and Kyoto encyclopedia of genes and genomes (KEGG) pathway enrichment analysis of *NtGCN2* overexpression (OE) versus K326 under cold conditions. **(A–C)** Top 30 significantly enriched GO terms in Biological Process, Cellular Component, and Molecular Function categories at 0 h **(A)**, 3 h **(B)**, and 6 h **(C)** after cold exposure.**(D–F)** Top 20 enriched KEGG pathways identified at 0 h **(D)**, 3 h **(E)**, and 6 h **(F)** after cold exposure. Categories are indicated on the right: Environmental Information Processing (EnvI P), Genetic Information Processing (GenIP), Metabolism (Metab), and Organismal Systems (Organs).

Functional enrichment gene ontology (GO) revealed that DEGs under control conditions were enriched in 1170 GO terms. These included response to salicylic acid, positive regulation of auxin-mediated signaling pathways, and DNA-binding transcription factor activity (**Figure 5A**). Moreover, after 3 h of cold stress, enrichment shifted to 1417 terms. This was dominated by the positive regulation of auxin signaling, response to salicylic acid, the CCAAT-binding factor complex, and transcription regulatory region DNA binding (**Figure 5B**). After 6 h of cold stress, enrichment peaked at 1482 terms, with key categories implicated in cellular oxidant detoxification, regulation of response to osmotic stress, (+)-abscisic acid D-glucopyranosyl ester transmembrane transport/transporter activity, and dioxygenase activity (**Figure 5C**).

Pathway analysis (Kyoto Encyclopedia of Genes and Genomes) showed 72 enriched pathways under control conditions, notably peroxisomes, plant hormone signal transduction, and mitogen-activated protein kinase (MAPK) signaling pathway (**Figure 5D**). After 3 h of cold stress, 89 pathways were enriched, including plant hormone signal transduction, protein processing in the endoplasmic reticulum, phenylalanine/tryptophan metabolism, and alpha-linolenic acid metabolism (**Figure 5E**). After 6 h of cold stress, 88 pathways were enriched, primarily plant hormone signal transduction, circadian rhythm-plant, brassinosteroid biosynthesis, biosynthesis of various plant secondary metabolites, and alpha-linolenic acid metabolism (**Figure 5F**).

Validation was conducted via qRT-PCR. To confirm the RNA-seq reliability, six DEGs (*Nitab4.5_0005212g0050, Nitab4.5_0002419g0020, Nitab4.5_0021312g0010, Nitab4.5_0000222g0080 and Nitab4.5_0003878g0020, Nitab4.5_0003983g0070*) were analyzed. Their expression patterns under both control and cold-stress conditions were highly consistent with the RNA-seq data, thus validating the transcriptomic findings (**Supplementary Figure S2**).

### 3.5 Integrated analysis reveals molecular mechanisms of *NtGCN2*-mediated cold tolerance

**Figure 6:**
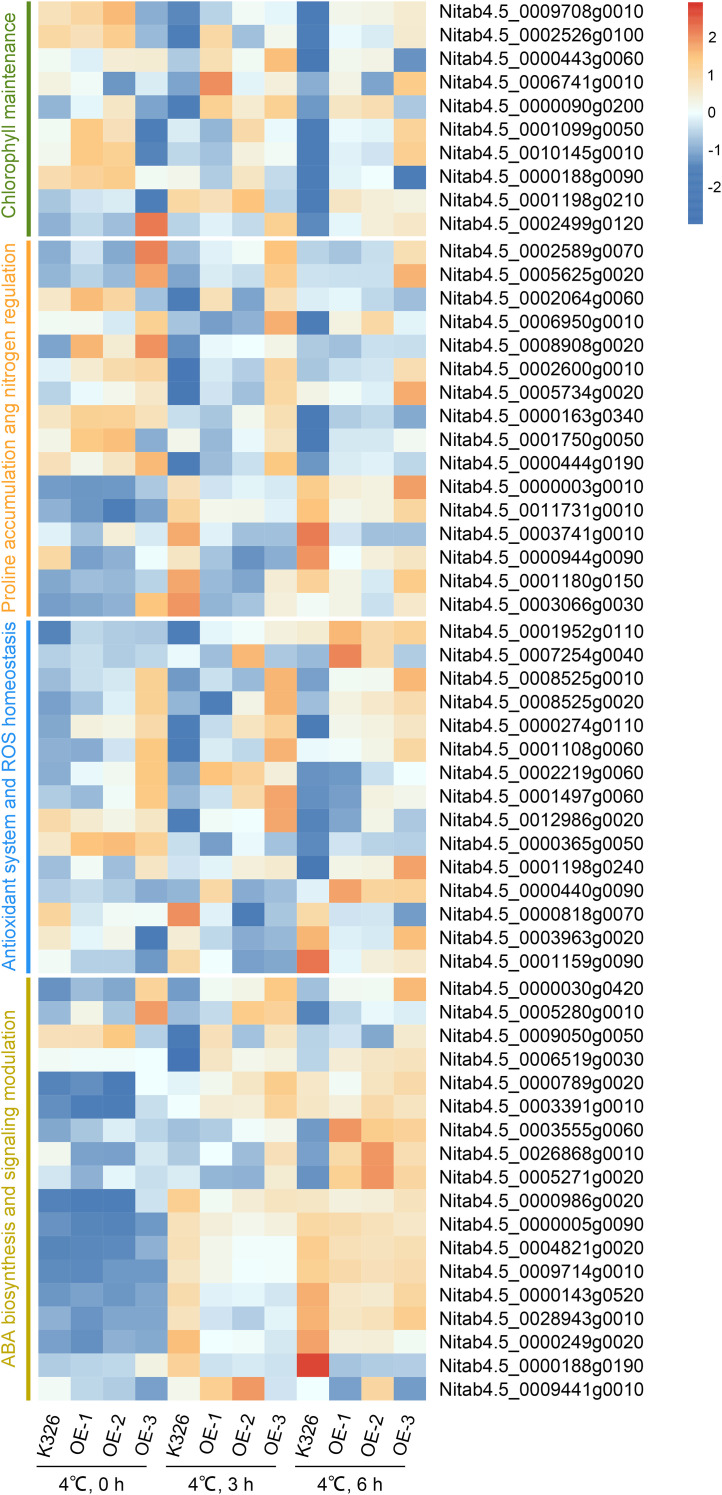
Heatmap representation of DEGs related to cold stress responses in transgenic (OE) and K326 tobacco plants exposed to cold stress for 0, 3, and 6 hours. Genes were grouped into four functional categories: Chlorophyll maintenance (green), Proline accumulation and nitrogen regulation (orange), Antioxidant system and ROS homeostasis (blue), and ABA biosynthesis and signaling modulation (yellow). Gene expression values were normalized and log_2_-transformed.

By integrating physiological, biochemical, and transcriptomic data, the molecular mechanisms through which *NtGCN2* confers cold tolerance were elucidated. The first mechanism was chlorophyll maintenance. Cold stress induced differential expression of 10 chlorophyll-related genes in OE plants. The upregulated genes included chlorophyll synthesis regulators [*HEMA1 (Nitab4.5_0009708g0010), POR1 (Nitab4.5_0002526g0100)*] and light signaling/chloroplast development factors [*FAR1 (Nitab4.5_0000443g0060), GRF5 (Nitab4.5_0006741g0010), PGR3 (Nitab4.5_0000090g0200), EGY3 (Nitab4.5_0001099g0050, Nitab4.5_0010145g0010), ATJ20 (Nitab4.5_0000188g0090), STY17 (Nitab4.5_0001198g0210), CRR2 (Nitab4.5_0002499g0120)*, and *HY5 (Nitab4.5_0009676g0030)*]. This coordinated upregulation provides a transcriptional basis for the observed preservation of chlorophyll levels in the OE lines under cold stress (**Figure 6**; **Supplementary Table S4**).

The second mechanism was proline accumulation and nitrogen regulation. *NtGCN2*-OE modulated the key genes involved in proline metabolism under cold stress. In terms of synthesis/accumulation, proline aminopeptidase homologs [*PAP1 (Nitab4.5_0002589g0070; Nitab4.5_0005625g0020)* and *PAP2 (Nitab4.5_0002064g0060)*] and the proline transporter *ProT* (*Nitab4.5_0006950g0010*) were upregulated. In terms of metabolic regulation, the phosphate starvation regulator *PHR1* (*Nitab4.5_0008908g0020*) was upregulated, potentially activating *ProDH2/P5CS1* (**Figure 6**; **Supplementary Table S4**).

In terms of nitrogen signaling, nitrogen transporters, such as *AMT1;3* (*Nitab4.5_0002600g0010; Nitab4.5_0005734g0020*), *NRT1.4* (*Nitab4.5_0000163g0340; Nitab4.5_0001750g0050*), *AMT2* (*Nitab4.5_0000444g0190*), *NRT1.9* (*Nitab4.5_0000003g0010; Nitab4.5_0011731g0010*), and *NRT3.2* (*Nitab4.5_0003741g0010*), were actively regulated. In terms of glutamate flux, the glutamate decarboxylases *GAD3* (*Nitab4.5_0000944g0090*) and *GAD4* (*Nitab4.5_0001180g0150*; *Nitab4.5_0003066g0030*) were downregulated. This diverted glutamate toward proline synthesis (**Figure 6**; **Supplementary Table S4**).

The third mechanism was enhanced antioxidant capacity. Consistent with the elevated enzyme activity, *NtGCN2* OE upregulated critical ROS-scavenging components under cold stress. The following peroxidases were upregulated: *DOX1* (*Nitab4.5_0001952g0110; Nitab4.5_0007254g0040*), *POD* (*Nitab4.5_0008525g0010*), and *PER54* (*Nitab4.5_0008525g0020*). The following ROS homeostasis regulators were upregulated: *BPA1* (*Nitab4.5_0000274g0110; Nitab4.5_0001108g0060*), *SPL4* (*Nitab4.5_0002219g0060*), *AARE* (*Nitab4.5_0001497g0060; Nitab4.5_0012986g0020*), *MYB4* (*Nitab4.5_0000365g0050*), *AOX3* (*Nitab4.5_0001198g0240*), and *ANP2* (*Nitab4.5_0000440g0090*). In terms of reduced glutathione degradation, *GGT3* (*Nitab4.5_0000818g0070*) and *Chac2* (*Nitab4.5_0003963g0020; Nitab4.5_0001159g0090*) were downregulated. This transcriptional reprogramming directly supported the observed increase in peroxidase activity and reduced oxidative damage (**Figure 6**; **Supplementary Table S4**).

The fourth mechanism was ABA biosynthesis and signaling modulation. *NtGCN2* profoundly altered ABA metabolism and signaling. In terms of enhanced biosynthesis, carotenoid pathway genes [*NCED1 (Nitab4.5_0000030g0420; Nitab4.5_0005280g0010), VDE1 (Nitab4.5_0009050g0050)*, and *Z-ISO (Nitab4.5_0006519g0030)]* and ABA signaling components *[PP2C5 (Nitab4.5_0000789g0020; Nitab4.5_0003391g0010), ABCG40 (Nitab4.5_0003555g0060), DCAF1 (Nitab4.5_0026868g0010)*, and *MED16 (Nitab4.5_0005271g0020)*] were upregulated. In terms of reduced degradation/inhibition, the *ABA hydroxylase encoding gene CYP707A4* (*Nitab4.5*0000986g0020) and the ABA synthesis inhibitory transcription factor LHY (*Nitab4.5_0000005g0090; Nitab4.5_0004821g0020; Nitab4.5_0009714g0010*) was downregulated (**Figure 6**; **Supplementary Table S4**).

In terms of the attenuated signaling response, key signaling components, including *COP1* (*Nitab4.5_0000143g0520; Nitab4.5_0028943g0010*), *SDIR1* (*Nitab4.5_0000249g0020*), *DPBF4* (*Nitab4.5_0000188g0190*), and *RHA2B* (*Nitab4.5_0009441g0010*) were downregulated. These changes collectively explain the elevated ABA accumulation in the OE lines and suggest that *NtGCN2* fine-tunes ABA signaling during cold adaptation (**Figure 6**; **Supplementary Table S4**).

**Figure 7:**
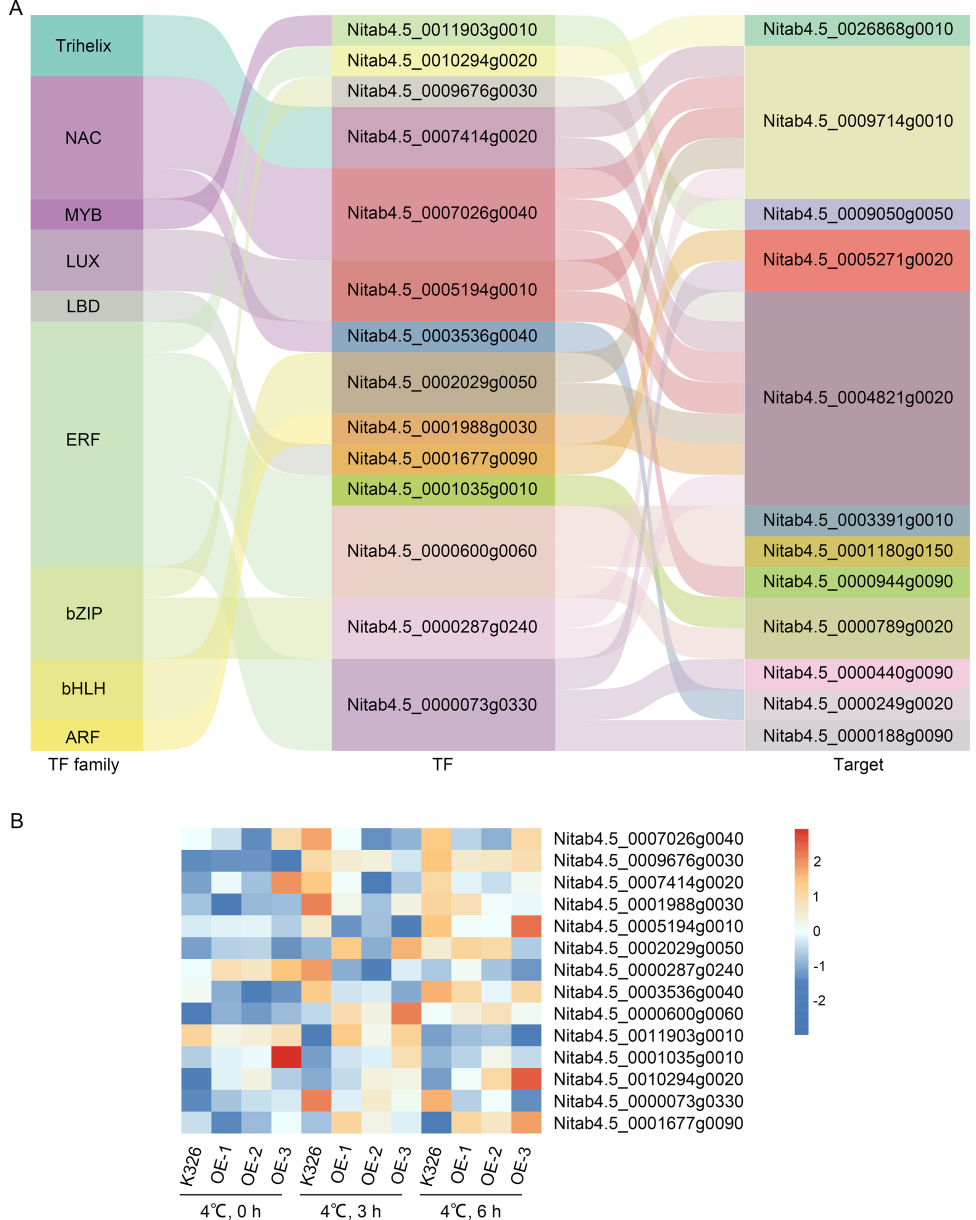
Transcription factor (TF)–target regulatory network and expression patterns under cold stress conditions. **(A)** Sankey diagram illustrating the associations between transcription factor families (left), individual transcription factors (middle), and their corresponding target genes (right) in tobacco under cold stress. TFs are classified into major families including Trihelix, NAC, MYB, LUX, LBD, ERF, bZIP, bHLH, and ARF. Links represent predicted or validated regulatory relationships. **(B)** Heatmap showing the expression profiles of selected TFs in wild-type (K326) and transgenic (OE) tobacco lines under low temperature treatment (4 °C) at 0, 3, and 6 hours.

To further investigate the transcriptional regulatory mechanisms under cold stress, differentially expressed transcription factors (TFs) were identified at multiple time points following low temperature treatment, and their potential target genes were predicted to construct a TF–target regulatory network. The analysis revealed several key TFs from different families, including *MYB*, *NAC*, *ERF*, and *bZIP*, which may regulate cold-responsive genes(**Figure 7A**). Notably, expression profiling showed that *ERF009* (*Nitab4.5_0010294g0020*) and *ERF095* (*Nitab4.5_0000073g0330*) were continuously upregulated under cold stress, whereas *NAC062* (*Nitab4.5_0007026g0040*), *HY5* (*Nitab4.5_0009676g0030*) and *ERF012* (*Nitab4.5_0000073g0330*) were consistently downregulated, suggesting distinct roles in the transcriptional response to cold exposure (**Figure 7B**; **Supplementary Table S5**).

## 4 Discussion

Cold conditions severely restrict plant growth, development, and distribution, resulting in severe yield loss and mortality (Gong et al., 2020; Soualiou et al., 2022; Zhang et al., 2022). The protein kinase GCN2 regulates stress adaptation across kingdoms by phosphorylating eIF2α to modulate protein synthesis; however, its specific mechanisms in plant cold responses remain poorly defined. Building on previous findings that *NtGCN2* enhances antioxidant capacity, ABA/proline accumulation, and growth (Li et al., 2018), this study integrated physiological, biochemical, and transcriptomic analyses to elucidate the mechanisms through which *NtGCN2* confers cold tolerance to tobacco. The study demonstrates that *NtGCN2* orchestrates a multi-faceted defense by upregulating genes involved in antioxidant defense, proline biosynthesis, ABA synthesis, and chlorophyll maintenance, thereby enhancing ROS scavenging, osmotic adjustment, stress signaling, and photosynthetic resilience under cold stress (4°C).

### 4.1 Chlorophyll preservation and photosynthetic integrity

Cold stress disrupts photosynthesis, notably through chlorophyll degradation and chloroplast impairment, manifesting as leaf yellowing and wilting. In this study, *NtGCN2* sustained the chlorophyll content by upregulating key biosynthesis genes (*HEMA1 and POR1*), chloroplast development/function genes (*GRF5, PGR3, EGY3, ATJ20, STY17*), and light-signaling components (*FAR1, COP1, SPA2, HY5*). This transcriptional program likely counteracts cold-induced chlorophyll catabolism and chloroplast dysfunction. This preserves photosynthetic capacity, which is a critical determinant of cold tolerance (Yuan et al., 2018; Gu et al., 2021; Li et al., 2024; Feng et al., 2025). Notably, upregulation of phyA signaling genes (*SCL21, FAR1*) suggests potential crosstalk between *NtGCN2* and light signaling pathways in stress adaptation, warranting further investigation.

### 4.2 Proline accumulation and osmotic regulation

To mitigate cold-induced cellular dehydration, plants accumulate osmolytes, such as proline (Hayat et al., 2012). *NtGCN2* overexpression markedly elevated proline levels. This correlated with upregulated proline biosynthesis (*PAP1, PAP2*) and transport *(AAP4, ProT)* genes and downregulated glutamate decarboxylase (*GAD3, GAD4*), thereby diverting glutamate flux toward proline synthesis. Furthermore, *NtGCN2* modulates nitrogen transporter genes (*AMT1;3, AMT2, NRT1.4, NRT1.9, NRT3.2*), thus implicating nitrogen signaling in osmotic adjustment. Proline accumulation is also regulated by ABA, ROS (H_2_O_2_), and Ca²^+^ signaling (Yuan et al., 2018; Shrestha et al., 2021; Li et al., 2022). The study data suggest that *NtGCN2* may indirectly potentiate proline accumulation by elevating ABA levels. This modulates ROS homeostasis and upregulates Ca²^+^ signaling components (*RGA1/COLD1, CIPKs, CMLs, CNGCs*), ultimately forming an integrated osmotic defense network.

### 4.3 Antioxidant defense against oxidative stress

Cold-induced ROS imbalance causes oxidative damage (Zhou et al., 2025). *NtGCN2* mitigated this effect by enhancing SOD and CAT activities and upregulating peroxidase genes (*DOX1, POD, PER54*) and ROS homeostasis regulators (*BPA1, SPL4, AARE, MYB4, AOX3, ANP2*), while suppressing glutathione degradation (*GGT3, Chac2*). This coordinated upregulation of antioxidant defenses, consistent with previous studies on drought tolerance, directly scavenges excess ROS and protects cellular integrity under cold-stress conditions.

### 4.4 ABA Biosynthesis and signaling dynamics

ABA is a central regulator of cold acclimation (Chen et al., 2020). *NtGCN2* substantially elevated ABA levels by upregulating carotenoid pathway genes (*NCED1, VDE1, Z-ISO*) and downregulating the ABA catabolic enzyme encoding gene *CYP707A4* and the negative transcription factor *LHY*. Intriguingly, *NtGCN2* simultaneously downregulated key ABA-signaling components (*COP1, SDIR1, DPBF4, RHA2B*) and promoted ABA accumulation under cold stress. This apparent decoupling of high ABA levels from downstream signaling attenuation suggests that *NtGCN2* may fine-tune ABA sensitivity or utilize post-transcriptional regulation to modulate stress responses. The elevated basal ABA in *NtGCN2*-OE plants under control conditions, despite transcriptomic evidence suggesting suppressed synthesis and enhanced degradation, further implies the complex post-transcriptional or post-translational control of ABA homeostasis by *NtGCN2*.

While this study provides valuable insights into the role of *NtGCN2* in cold tolerance, several limitations should be acknowledged. First, the conclusions are primarily based on overexpression lines, without validation from loss-of-function mutants to fully confirm gene-specific effects. Second, the proposed regulatory framework—linking *NtGCN2* to photosynthetic maintenance, osmotic adjustment, ROS scavenging, and ABA accumulation-is largely inferred from integrated physiological and transcriptomic analyses. Further validation using genetic and molecular approaches, such as loss-of-function mutants, protein-DNA interaction assays, and post-transcriptional regulatory analyses, will be required to confirm these mechanisms. Finally, although transcriptome profiling revealed extensive reprogramming of stress-responsive pathways, the functional roles of key downstream targets, including *NCED1* in ABA biosynthesis and *PAP1* in proline metabolism, remain to be experimentally validated. Clarifying their specific contributions, as well as their potential involvement in coordinating multiple processes such as ROS homeostasis, Ca²^+^ signaling, and light-responsive pathways, will be essential for refining the proposed model. Collectively, these future studies will be critical for establishing a more comprehensive and mechanistic understanding of how *NtGCN2* integrates multiple regulatory networks to confer cold tolerance in plants.

## 5 Conclusion

**Figure 8:**
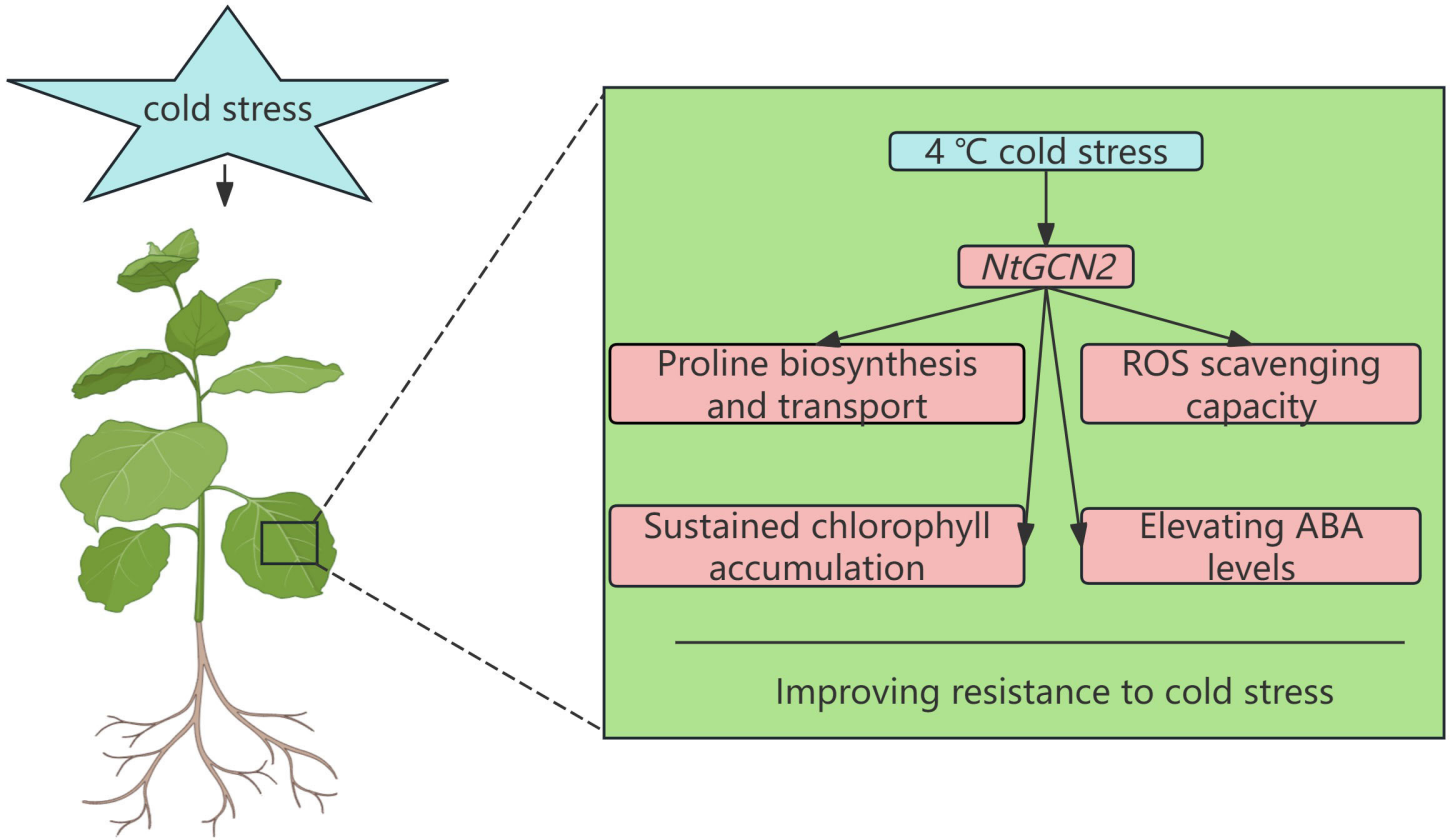
Proposed model for *NtGCN2*-mediated cold stress tolerance. By integrating physiological, biochemical, and transcriptomic data, the study proposed a mechanistic model wherein *NtGCN2* orchestrates cold adaptation as follows: (1) modulating osmotic balance through enhanced proline biosynthesis and transport, (2) ameliorating oxidative damage by upregulating ROS scavenging capacity; (3) maintaining photosynthetic integrity via sustained chlorophyll accumulation, and (4) elevating ABA levels to potentiate stress signaling.

This study established a critical role for *NtGCN2* in conferring cold tolerance in tobacco. The study demonstrated that *NtGCN2* overexpression enhanced plant growth vigor, bolstered antioxidant capacity, promoted ABA biosynthesis, and preserved chlorophyll content under low-temperature stress. By integrating physiological, biochemical, and transcriptomic data, the study proposed a mechanistic model wherein *NtGCN2* orchestrates cold adaptation as follows: (1) modulating osmotic balance through enhanced proline biosynthesis and transport, (2) ameliorating oxidative damage by upregulating ROS scavenging capacity; (3) maintaining photosynthetic integrity via sustained chlorophyll accumulation, and (4) elevating ABA levels to potentiate stress signaling (**Figure 8**). Collectively, these findings confirmed that *NtGCN2* enhances cold tolerance by regulating proline metabolism, antioxidant defense, chlorophyll homeostasis, and ABA biosynthesis. This study considerably expands the functional understanding of the conserved GCN2 signaling network in plants and provides valuable genetic targets for improving crop resilience to cold stress. Further research should investigate the upstream signaling mechanisms that activate *NtGCN2* under cold stress, including its potential regulation by amino acid availability, tRNA phosphorylation, or other stress-induced cues. Additionally, exploring the cross-talk between *NtGCN2* and other stress response pathways—such as ABA signaling, ROS homeostasis, and cold-responsive transcriptional networks—will be essential for elucidating its integrative role in plant stress adaptation.

## Data Availability

The datasets presented in this study can be found in online repositories. The names of the repository/repositories and accession number(s) can be found in the article/**Supplementary Material**.
