# Supplementary material for: NtGCN2 mediates tobacco cold tolerance through chlorophyll retention, proline accumulation, antioxidant defense, and ABA regulation
Source: Front Plant Sci. 2026 May 8;17:1833586. doi: 10.3389/fpls.2026.1833586 (PMC13196610; doi:10.3389/fpls.2026.1833586)
Supplement: Supplementary file 2 [file Table2.docx]

**Supplementary Figure**

**
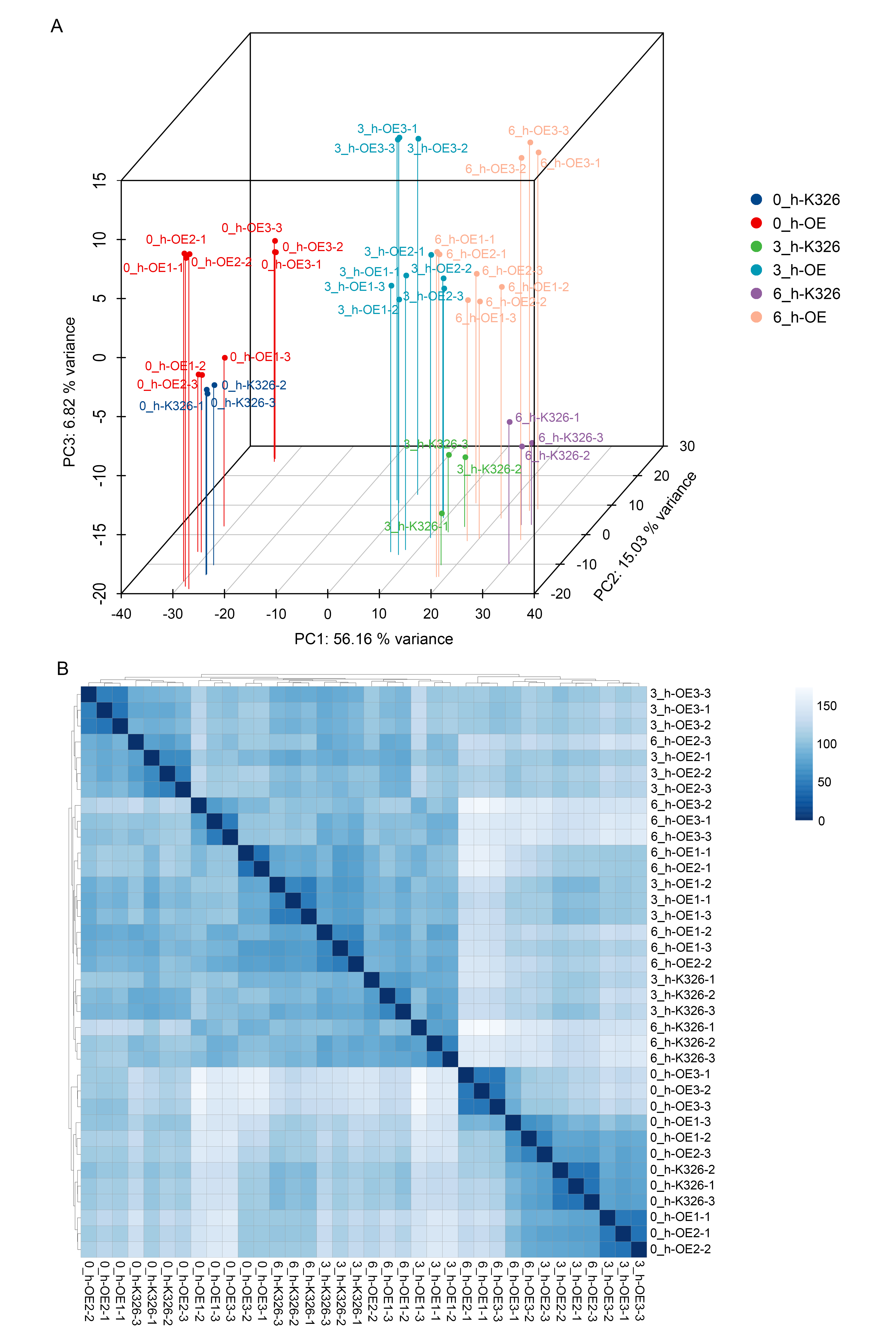
**

**Fig. S1. Between-sample principal component analysis and heatmap of cluster analysis.** (A)Three-dimensional PCA was performed using FPKM values of all expressed genes to explore the global transcriptomic variation among samples. Each point represents one biological replicate from the six groups (ZW, ZOE, TW, TOE, SW, and SOE). The first three principal components (PC1, PC2, and PC3) explain 56.16%, 15.03%, and 6.82% of the total variance, respectively. Samples from the same group cluster closely together, indicating high intra-group similarity and distinct transcriptional signatures across different tissue types. (B) Pairwise Euclidean distances between all biological replicates were calculated using normalized FPKM values and visualized as a heatmap. Both rows and columns are hierarchically clustered to illustrate the relationships among samples across six tissue groups (ZW, ZOE, TW, TOE, SW, and SOE). Clear clustering of replicates within each group highlights the consistency of biological replicates and distinct gene expression patterns across tissue types.


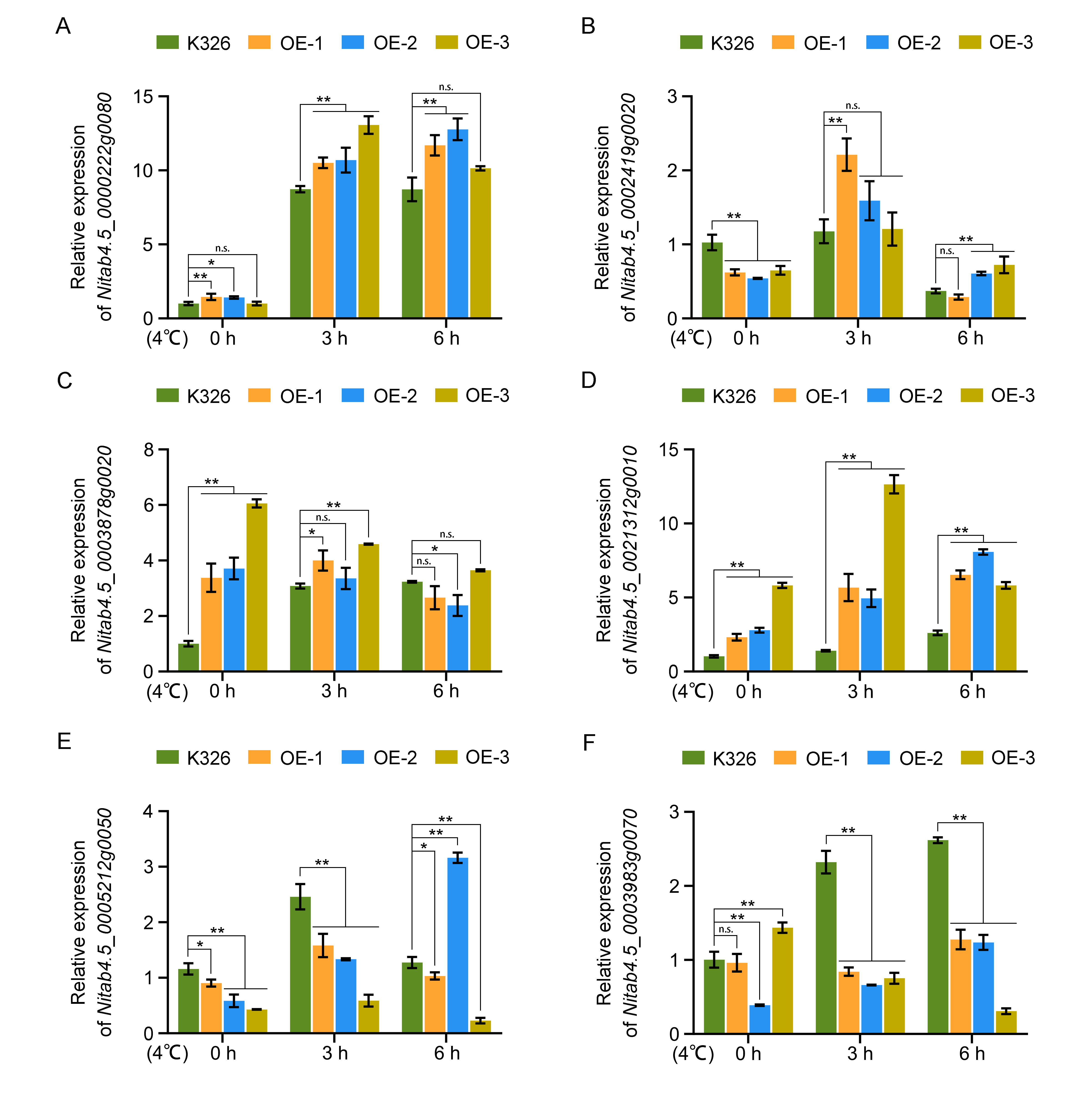


**Fig. S2. Verification of transcriptomic data using quantitative qRT-PCR.**

qRT-PCR analysis of DEGs including *Nitab4.5_0000222g0080* (A), *Nitab4.5_0002419g0020* (B), *Nitab4.5_0003878g0020* (C), *Nitab4.5_0021312g0010* (D), *Nitab4.5_0005212g0050* (E) and *Nitab4.5_0003983g0070* (F) in transgenic and K326 plants under cold treatment for 0, 3, 6 hours. In (A-F), error bars represent the means ± SD (n = 3) taken from three independent biological replicates. Asterisks indicate a significant difference from K326’s at * *P* < 0.05 or ** *P* < 0.01 by the Duncan test.
